# Hypoxia mediated pulmonary edema: potential influence of oxidative stress, sympathetic activation and cerebral blood flow

**DOI:** 10.1186/s12899-015-0018-4

**Published:** 2015-10-09

**Authors:** Shadi Khademi, Melinda A. Frye, Kimberly M. Jeckel, Thies Schroeder, Eric Monnet, Dave C. Irwin, Patricia A. Cole, Christopher Bell, Benjamin F. Miller, Karyn L. Hamilton

**Affiliations:** Department of Health and Exercise Science, Colorado State University, Fort Collins, CO 80523 USA; Department of Biomedical Sciences, Colorado State University, Fort Collins, CO 80523 USA; Department of Radiation Oncology, Duke University Medical Center, Durham, NC USA; Clinical Sciences, Colorado State University, Fort Collins, CO 80523 USA; Cardiovascular Pulmonary Research, University of Colorado Denver, Anschutz Medical Campus, Denver, CO 80045 USA; Department of Microbiology, Immunology and Pathology, Colorado State University, Fort Collins, CO 80523 USA; 3333 Burnet Avenue, Building R, Room 3503, Cincinnati, 45229 OH USA

**Keywords:** Cerebral hypoxia, Nrf2, HO-1, Catecholamines, Cerebral autoregulation

## Abstract

**Background:**

Neurogenic pulmonary edema (NPE) is a non-cardiogenic form of pulmonary edema that can occur consequent to central neurologic insults including stroke, traumatic brain injury, and seizure. NPE is a public health concern due to high morbidity and mortality, yet the mechanism(s) are unknown. We hypothesized that NPE, evoked by cerebral hypoxia in the presence of systemic normoxia, would be accompanied by sympathetic activation, oxidative stress, and compensatory antioxidant mechanisms.

**Methods:**

Thirteen Walker hounds were assigned to cerebral hypoxia (SaO_2_ ~ 55 %) with systemic normoxia (SaO_2_ ~ 90 %) (CH; *n* = 6), cerebral and systemic (global) hypoxia (SaO_2_ ~ 60 %) (GH; *n* = 4), or cerebral and systemic normoxia (SaO_2_ ~ 90 %) (CON; *n* = 3). Femoral venous (CH and CON) perfusate was delivered via cardiopulmonary bypass to the brain and GH was induced by FiO_2_ = 10 % to maintain the SaO_2_ at ~60 %. Lung wet to lung dry weight ratios (LWW/LDW) were assessed as an index of pulmonary edema in addition to hemodynamic measurements. Plasma catecholamines were measured as markers of sympathetic nervous system (SNS) activity. Total glutathione, protein carbonyls, and malondialdehyde were assessed as indicators of oxidative stress. Brain and lung compensatory antioxidants were measured with immunoblotting.

**Results:**

Compared to CON, LWW/LDW and pulmonary artery pressure were greater in CH and GH. Expression of hemeoxygenase-1 in brain was higher in CH compared to GH and CON, despite no group differences in oxidative damage in any tissue. Catecholamines tended to be higher in CH and GH.

**Conclusion:**

Cerebral hypoxia, with systemic normoxia, is not systematically associated with an increase in oxidative stress and compensatory antioxidant enzymes in lung, suggesting oxidative stress did not contribute to NPE in lung. However, increased SNS activity may play a role in the induction of NPE during hypoxia.

## Background

Neurogenic pulmonary edema (NPE) is an acute pulmonary edema that develops after a central neurologic insult, and is believed to occur independent of a cardiogenic contribution [1]. NPE can occur following stroke, traumatic brain injury, and seizure, and represents a major public health concern due to high morbidity (40-50 %) and mortality (7 %) [2]. Currently, the underlying mechanism(s) of NPE are poorly understood. However, in animal models increased intracranial pressure (ICP) coupled to increased sympathetic nervous system (SNS) activation is strongly linked to development of NPE [3, 4]. In addition to increased ICP and SNS activation, cerebral hypoxia is a common feature of many neuropathologies such as stroke, brain injury, and seizure [5], suggesting that cerebral hypoxia may be an important factor in the pathogenesis of NPE.

In the past four decades only a handful of studies have sought to investigate the mechanistic link between cerebral hypoxia and the induction of pulmonary edema. Between 1971–1976, Moss and Stein pioneered a canine model in which cerebral hypoxia was induced by directing venous perfusate to the brain in the presence of pulmonary and systemic normoxia, resulting in pulmonary edema [6–9]. Although their work showed that cerebral hypoxia can lead to pulmonary edema, the authors only speculated on a mechanism, which was thought to be hydrostatic in origin from increased pulmonary venous resistance [6–9]. More recently, our group confirmed and extended the findings of the Moss model to show cerebral hypoxia-induced pulmonary edema occurred via a mechanisms other than increased hydrostatic pressure in the pulmonary vasculature [10]. However, in our previous experiment, alterations in ICP, cerebral blood flow (CBF), or reactive oxygen species (ROS), were not assessed as potential mechanisms of altered lung permeability that lead to pulmonary edema. It is plausible that cerebral hypoxia contributes to pulmonary edema not only through a hypoxic pulmonary vasoconstrictive effect but also by increasing ICP, which subsequently triggers increased SNS activation [4]. In animal models of NPE there is pronounced activation of the SNS as a necessary prerequisite for the development of NPE [11]. Increased ICP due to hypoxia could be caused by either brain tissue swelling or increased CBF to the brain to counteract brain tissue hypoxia [12, 13].

Cerebral hypoxia also promotes generation of ROS, leading to increased oxidative damage to lipids, proteins, and DNA [14]. There is an established link between increased oxidative stress and the pathogenesis of an array of human diseases [15] including a potential role of oxidants in the pathogenesis of acute lung injury [16–18]. It is therefore possible that cerebral hypoxia induces excessive circulating brain derived ROS that impair pulmonary endothelial barrier function and contribute to the development of NPE.

The endogenous cellular antioxidant defense system consists of approximately 400 proteins [19]. The transcription factor nuclear factor erythroid 2 related factor 2 (Nrf2) is the master regulator of endogenous cellular antioxidant defenses [20]. Through transcriptional activation of an antioxidant response element (ARE) bearing genes involved in antioxidant defenses, such as the phase II antioxidants superoxide dismutase 1 (SOD1), NAD (P) H: quinone oxidoreductase 1 (NQO1), and heme oxygenase 1 (HO-1), Nrf2 activates cellular pathways that protect against oxidative injury [20–22].

Nrf2 can be activated by oxidants produced during hypoxia [23]. Further, it has been shown that HO-1 increases in the rostral ventrolateral medulla during chronic hypoxia and is associated with cardiorespiratory adaptations to hypoxia [24]. Sharma et al. showed that during hypoxia, SOD1 expression is increased in the cortex to neutralize the hypoxia-induced ROS generation [25]. Given the evidence of ROS generation during hypoxia, and based on studies that have suggested a role for oxidative stress in induction of pulmonary edema, we investigated whether or not oxidative stress and compensatory antioxidants (HO-1, NQO1 and SOD1) play a role in both cerebral and global hypoxia-induced pulmonary edema.

Herein, based on our previous work demonstrating that cerebral hypoxia-induced pulmonary edema was not due to a hydrostatic origin [10], we hypothesized that pulmonary edema, evoked by cerebral hypoxia in the presence of systemic normoxia, is associated with increased ICP, SNS activation, and increased oxidative stress. In the present study, we designed experiments to monitor ICP, control CBF, and analyze markers for oxidative stress in both the brain and lung utilizing both cerebral-hypoxia induced NPE and global hypoxia-induced pulmonary edema models.

## Methods

### Experimental design

All surgical procedures and methods were approved by Colorado State University Animal Care and Use Committee (protocol numbers: 09-1351A and 11-2663A) and adhered to the Guide for the Care and Use of Agricultural Animals in Agricultural Research and Teaching. Thirteen male adult Walker hounds (25 – 30 kg), 4 years of age, were housed in individual stalls (Veterinary Teaching Hospital of Colorado State University, Fort Collins, Colorado, USA) , provided water and food ad libitum in 5000 feet of altitude, were adapted to these conditions for at least 1 week before being used in the experiments, and randomly assigned to one of three groups: (1) control (CON, *n* = 3), cerebral normoxia with systemic normoxia (SaO_2_ ~ 90 %); (2) cerebral hypoxia (CH, *n* = 6), cerebral hypoxia (SaO_2_ ~ 55 %) with systemic normoxia (SaO_2_ ~ 90 %); (3) global hypoxia (GH, *n* = 4), systemic hypoxia (SaO_2_ ~ 60 %). All efforts were made to minimize the suffering and the number of animals. This work is written according to the ARRIVE guidelines (http://www.nc3rs.org.uk/ARRIVE/).

### Instrumentation

Anesthetic and surgical procedures were adapted from those previously described methods [10] and reflecting a modification to the original model developed by Moss and Stein [9]. Following an overnight fast, the dogs were sedated with fentanyl (10 μg/kg SQ), atropine (0.04 mg/kg SQ) and midazolam (0.2 mg/kg SQ) 30 min prior to anesthetic induction. Anesthesia was induced by propofol (2.5 mg/kg IV) and maintained by fentanyl (0.01 mg/kg IV). Once a surgical plane of anesthesia was confirmed, animals in the GH group were intubated and placed on a volume ventilator (10 breaths/minute) with 100 % oxygen and isoflurane (1.0-2.0 %) during surgical isolation of the jugular veins, carotid, and femoral arteries. A catheter was placed underneath the meninges to estimate intracranial pressure (ICP). ICP was recorded every 15 min on a pressure monitor (Marquet 7000, Fridley, MN). Cerebral perfusion pressure (CPP) (mmHg) was calculated by subtracting the ICP (mmHg) from the mean arterial pressure (MAP) (mmHg). Cardiac output (CO) was measured by thermodilution method [26] with 10 mL of iced saline. Pulmonary arterial pressure (PAP) and capillary wedge pressure (Pwedge) were assessed using a Swan-Ganz catheter inserted into the pulmonary artery. A catheter was placed in a dorsal pedal artery to measure MAP. Stroke volume (SV) and systemic vascular resistance (SVR) were calculated from the quotients of CO (L/min) divided by HR (bpm), and MAP divided by CO respectively. Pulmonary vascular resistance (PVR) was calculated by the subtraction of Pwedge from PAP divided by CO ((PAP-Pwedge)/CO) and is presented as the product of dyne and time relative to cm5 (dyn*s/cm5). A 2 mm flow probe (Transonic, Transonic Systems, Inc., Ithaca, NY) was placed around each carotid artery to measure blood flow. Data were collected at baseline and then every 30 min during the experiment.

### Induction of cerebral or global hypoxia

Dogs assigned to the cerebral hypoxia cohort had their left femoral vein cannulated with a 14 Fr cannula and connected to the inlet port of a cardiopulmonary bypass pump (Roller pump, Cobe, Lakewood, CO, USA). Both carotid arteries were cannulated with an 8 Fr cannula and connected to the pump outlet with a “Y” connector. When venous and arterial isolations were achieved, the animals were weaned to 21 % O2 and ventilation rate adjusted to 8 - 10 breaths/minute to maintain normal systemic partial pressure of CO2 and oxygen saturation (SaO_2_ ~ 95 %). To maintain control over cerebral hypoxic blood flow and insure oxygenated arterial blood was not entering the brain via the vertebral arteries, both vertebral arteries were isolated at the level of 7th cervical vertebrae and occluded by tourniquets during the time course of cerebral hypoxia.

After normal carotid blood flow was achieved from the cardiopulmonary bypass pump, carotid arteries were perfused with femoral venous blood desaturated to 60 % for the CH group or saturated to ~95 % for the CON group. Carotid arterial blood flow of the CH cohort was matched to the blood flow of the GH cohort. Carotid arterial blood flow in the CON group was clamped at baseline values obtained prior to cannulation of carotid arteries. All animals underwent 2 hours of hypoxia (or normoxia in the CON group).

As described above, once a surgical plane of anesthesia with isoflurane (1.0-2.0 %) was confirmed, animals in the GH group were intubated and placed on a volume ventilator (10 breaths/minute). Dogs received 10 % O2 and when necessary room air was introduced to maintain 60 %.

### Blood and tissue acquisition

Blood (6 mL) was drawn from the dorso-pedal arterial and jugular venous and internal carotid catheters at baseline, 30, 60, 90 and 120 min of hypoxia or normoxia. Samples were transferred into chilled vials, one set containing ethylenediaminetetraacetic acid (EDTA; 1.8 mg K3 EDTA per 1 mL of blood) and the other set containing EDTA, 0.3 M ethylene glycol tetraacetic acid (EGTA), 0.3 M glutathione. Plasma was separated by centrifugation (4 °C, 14,000 x g; 10 min), and stored at -80 °C until assayed. At the end of 2 hours of hypoxia/normoxia, the animals were euthanized with sodium pentobarbital (10 mL IV) and the heart and lungs were removed by median sternotomy. The left caudal lung lobe was removed, weighed, and oven dried (65 °C for 96 hours until stable weight was achieved) for lung wet weight to lung dry weight (LWW/LDW) ratios as indices of pulmonary edema. The right caudal lobe was frozen in liquid nitrogen and stored at -80 °C for further analysis. Remaining lung lobes were fixed in 10 % formalin for 24 hours, placed into 70 % ethanol, paraffin embedded and cut into 4 µm sections for histological analyses. Brainstem, cerebellum, and left half of the cerebral cortex, were removed, snap frozen in liquid nitrogen, and stored at -80 °C for later analysis. A coronal section through the cortex around the prefrontal lobe was prepared for histological analyses in a manner identical to that used for lung tissue.

### Western blot analyses

Approximately 50 mg of frozen brain cortex and lung were homogenized (Next Advance Inc, Averill Park, NY, USA) in 1 mL of ice-cold buffer (40 mM Tris HCl, 10 mM Tris Base, 5 mM MgCl2, 100 mM NaCl, 1 % TritonX-100, 1 mM EDTA, pH 7.4) with protease and phosphatase inhibitors (Halt, Thermo Fisher, Rockford, IL, USA). Samples were centrifuged (4 °C, 10,000 x g, 10 min) and protein concentration of the supernatant was determined using a bicinchoninic acid assay (Thermo Fisher, Rockford, IL, USA). Samples were heat denatured in Laemmli buffer, separated using 10 % SDS-PAGE, transferred to nitrocellulose paper, and incubated in 5 % milk in TBST (Tris-buffered saline with tween) for 1 hour prior to immunoblotting. To prepare the nuclear extracts, 40 mg of cerebral cortex and lung tissue were homogenized in 200 volumes of Thermo Scientific NE-PER Nuclear and Cytoplasmic Extraction Kit buffer (Thermo Fisher, Rockford, IL, USA). 20 µg of the nuclear protein (lung and brain) was then prepared for immunoblotting as described above. Antibodies were purchased from Abcam (Cambridge, MA, USA; HO-1 # Ab13248, NQO1 # Ab2346) and Santa Cruz Biotechnology (Santa Cruz, CA, USA; Nrf2 #SC-1302, SOD1# SC-8637). Blots were incubated overnight at 4 °C with primary antibodies diluted 1:200 in TBST, washed in TBST, and incubated with HRP-conjugated secondary antibody diluted 1:1000 in 5 % milk in TBST for 1 hour at room temperature (anti-rabbit for Nrf2, anti-goat for SOD1 and NQO1 and anti-mouse for HO-1) followed by chemiluminescence detection (West Dura; Pierce, Rockford, IL, USA). Images were captured and densitometry conducted using a UVP Bioimaging system (Upland, CA, USA). Equal loading was verified using ponceau staining as well as actin antibodies (SC-8432; Santa Cruz Biotechnology, Santa Cruz, CA, USA).

### Immunohistochemistry (IHC)

Aforementioned 4 μm thick paraffin sections from brain cortex were mounted on poly-l-lysine slides. Slides were dewaxed and sections rehydrated by immersion in ethanol (100 %, 95 %, and 70 %) and then distilled water. After washing, sections were preincubated in PBS supplemented with 0.5 % BSA and 10 % normal horse serum (Santa Cruz Biotechnology, Santa Cruz, CA, USA) for 1 hour, then incubated overnight with mouse monoclonal anti-APPA4 antibody diluted 1:100 in PBS containing 0.5 % BSA and 15 % normal horse serum. The sections were then incubated for 1 hour with 1:1000 biotin-labeled anti-mouse secondary antibodies (Santa Cruz, CA, USA), followed by streptavidin-biotin-horseradish peroxidase solution containing  3′, 3′-diaminobenzidine (DAB) tetrahydrochloride dihydrate (Dakocytomation, CA, USA) and hydrogen peroxide. Finally, the sections were counterstained with hematoxylin. Signal density was quantified using Image J software (NIH, USA).

### Assessment of oxidative stress

Carbonylated proteins of lung and brain tissues were detected and analyzed following derivatization of protein carbonyl groups with 2, 4-dinitrophenylhydrazine, using the OxyBlot kit (Millipore, Billerica, MA, USA). Immunodetection was performed using 15 μg of protein per lane (3µg/µl) and primary antibody directed against dinitrophenylhydrazone (Millipore, Billerica, MA, USA). To measure malondialdehyde (MDA) concentrations, 25 mg of lung and brain tissue was quantitated using a TBAR assay kit (Cayman Chemical, Ann Arbor, MI, USA) per manufacturer recommendations. Plasma catecholamines: Plasma obtained from the blood that was collected into tubes with EGTA/glutathione was used to quantitate circulating norepinephrine and epinephrine with enzyme-linked immunosorbent assay (ELISA) (Rocky Mountain Diagnostics Inc., CO, USA).

### Statistical analyses

Hemodynamic data from all 13 subjects were included in analyses (CH=6, GH=4, CON=3). Unless otherwise noted, the remaining analyses were conducted using data from 12 dogs (tissues were not collected from the first CH procedure). Statistical analyses were performed with the SAS (version 9) statistical package (SAS, Cary, NC) and SPSS (IBM, version 19). Inter-subject variability in baseline hemodynamic measurements was controlled for by considering baseline measurements as covariates. Data were analyzed by one-way repeated measures of analysis of variance (ANOVA). Western blot, IHC, and catecholamine data were analyzed by one-way ANOVA. Significance was established a priori at p < 0.1 due to the necessary small sample size using this large animal model.

## Results

Oxygen saturation: To maintain the desired hypoxemic exposure to either cerebral or global hypoxia, or ensure a normal arterial oxygen saturation (SaO_2_), SaO_2_ was continuously monitored and recorded at 15 min intervals in all dogs. For dogs assigned to the cerebral hypoxia (CH) group, measurement of femoral and carotid SaO_2_ verified that cerebral hypoxia was established in the presence of systemic normoxia (SaO_2_ 54 ± 6 % carotid vs.94 ± 1.7 % femoral arterial blood; Table 1, *p* < 0.05). Global hypoxia (GH) elicited the expected lower SaO_2_ in both femoral and carotid arteries during the 2-h study period. Thus, we successfully induced and maintained both isolated cerebral hypoxia as well as global hypoxia.

**Table 1:** Arterial oxygen saturation (SaO_2_) of blood from femoral and carotid arteries in CON, CH and GH animals after 2 h of hypoxia

	Femoral artery SaO_2_	Carotid artery SaO_2_
CON	95.80 ± 3.42	95.60 ± 2.82
CH	94.50 ± 1.73	54.68 ± 6.27*
GH	61.95 ± 2.15**	60.00 ± 3.78*

*p < 0.05 compared to CON femoral artery and CON carotid artery, **p < 0.05 compared to CH femoral artery). Data presented as mean ± SEM

**Fig. 1:**
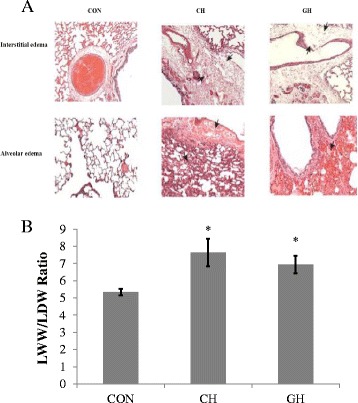
Lung histology and wet weight to dry weight (LWW/LDW) ratio. **a** Interstitial edema and areas of alveolar edema are presented in CON, CH, and GH on the top and bottom row respectively. **b** 2 h of CH and GH increased the LWW/LDW ratio, indicating increased pulmonary vascular leak. *p < 0.05 compared to CON. Data presented as mean ± SEM

Pulmonary edema: Assessment of the lung at necropsy, lung wet weight to dry weight (LWW/LDW) ratios, and histological analyses were used to evaluate the presence of pulmonary edema. Compared to controls (CON), the gross specimens of lungs showed more visible areas of petechiae and marked areas of interstitial and alveolar edema in the CH and GH cohorts (Fig. 1a) as well as greater LWW/LDW ratios (Fig. 1b, *p* < 0.05). These findings confirm that both isolated cerebral hypoxia and global hypoxia elicited the formation of pulmonary edema during the 2-h study period.

Hemodynamics: Hemodynamic measurements are presented in Table 2. Mean cardiac output (CO) at all time-points was higher in the GH (*p* = 0.07) compared to CH (*p* < 0.05) and CON (*p* = 0.07) cohorts. The higher CO in GH animals was attributed to a higher SV (*p* < 0.05) of GH animals. Our data show MAP was lower in CH compared to CON groups (*p* < 0.05), but no difference was noted between the CH and GH cohorts. Compared to CON cohorts, the systemic vascular resistance (SVR) was lower in GH and CH cohorts compared to CON (*p* < 0.1). Compared to the CON, pulmonary arterial pressure (PAP) at 2 h was greater in the GH and CH cohort (*p* < 0.1). Finally, Pwedge pressure was similar across groups.

**Table 2:** Hemodynamic measurements during experimentation. Data represent mean ± SEM of measures taken every 30 min under sham operated animals (CON), cerebral hypoxia (CH), and global hypoxia (GH). Baseline values were measured prior to induction of experimental conditions. The last column in the table represents the mean ± SEM of all time points considering baseline measurements as covariates

Measures	Groups	Baseline	30 min	60 min	90 min	120 min	Mean ± SEM at all time points
HR	CON	116 ± 5	118 ± 14	134 ± 19	122 ± 22	124 ± 9	119 ± 15
(bpm)	CH	111 ± 4	116 ± 12	107 ± 5	117 ± 10	124 ± 9	114 ± 10
	GH	90 ± 6	111 ± 12	98 ± 11	101 ± 13	103 ± 9	111 ± 15
MAP	CON	65 ± 6	78 ± 12	85 ± 1	82 ± 2	83 ± 8	86 ± 4
(mmHg)	CH	84 ± 11	82 ± 6	67 ± 6	74 ± 4	73 ± 6	73 ± 2*
	GH	81 ± 3	80 ± 2	77 ± 3	79 ± 2	77 ± 2	78 ± 3
PAP	CON	15 ± 1	14 ± 3	15 ± 1	14 ± 2	15 ± 1	15 ± 2
(mmHg)	CH	22 ± 4	22 ± 2	20 ± 2	23 ± 1	25 ± 1	23 ± 1*
	GH	22 ± 2	28 ± 2	24 ± 2	29 ± 3	29 ± 3	27 ± 2*
Pwedge	CON	11.7 ± 0.6	12.0 ± 0.6	11.7 ± 0.7	12.3 ± 1.2	12.3 ± 0.9	12.5 ± 0.9
(mmHg)	CH	14.2 ± 0.9	13.2 ± 0.8	13.0 ± 0.5	12.3 ± 0.9	12.5 ± 0.9	12.7 ± 0.5
	GH	15.8 ± 1.3	15.0 ± 1.2	13.8 ± 1.3	14.8 ± 0.3	15.0 ± 0.4	14.4 ± 0.7
CO	CON	2.6 ± 0.3	3.1 ± 0.8	3.3 ± 0.7	3.2 ± 0.9	3.7 ± 0.6	3.8 ± 0.5
(L/min)	CH	4.3 ± 1.2	4.3 ± 1.0	4.1 ± 0.7	4.1 ± 0.4	3.8 ± 0.3	3.9 ± 0.3
	GH	3.9 ± 0.4	5.4 ± 1.0	5.1 ± 0.4	5.2 ± 0.7	5.1 ± 0.7	5.2 ± 0.4*^#^
ICP	CON	12 ± 3	10 ± 2	11 ± 2	11 ± 3	11 ± 3	14 ± 2
(mmHg)	CH	21 ± 3	22 ± 3	21 ± 2	21 ± 3	22 ± 3	17 ± 1
	GH	11 ± 2	7 ± 2	10 ± 4	9 ± 3	11 ± 5	13 ± 2
SV	CON	23.3 ± 3.4	29.3 ± 11.5	26.8 ± 8.3	28.6 ± 9.5	34.1 ± 9.9	35.4 ± 4.3
(mL)	CH	40.8 ± 12.4	39.9 ± 8.7	39.7 ± 7.3	35.9 ± 3.5	31.7 ± 3.1	35.7 ± 2.9
	GH	44.8 ± 6.1	48.2 ± 3.9	53.2 ± 4.3	52.7 ± 7.0	50.2 ± 5.7	48.4 ± 3.6*^#^
PVR	CON	1.7 ± 0.9	3.1 ± 0.8	1.5 ± 0.4	2.6 ± 0.7	2.5 ± 0.5	2.4 ± 0.7
(dyn*s/cm^^5^)	CH	2.1 ± 0.5	3.6 ± 1.9	2.1 ± 0.6	2.6 ± 0.4	3.2 ± 0.6	2.8 ± 0.5
	GH	1.3 ± 0.2	1.1 ± 0.6	1.8 ± 0.5	1.3 ± 0.9	1.5 ± 0.7	1.6 ± 0.6
SVR	CON	24.8 ± 0.9	26.9 ± 4.1	29.6 ± 8.6	29.3 ± 6.5	24.3 ± 7.1	27.6 ± 3.2
(dyn*s/cm^^5^)	CH	27.2 ± 5.2	26.0 ± 7.3	18.3 ± 3.5	19.2 ± 2.7	19.5 ± 2.0	19.8 ± 2.3*
	GH	21.9 ± 3.7	16.0 ± 2.6	15.5 ± 1.7	16.4 ± 2.9	15.9 ± 2.8	17.3 ± 2.8*
CPP	CON	53 ± 10	68 ± 14	74 ± 3	71 ± 4	72 ± 6	74 ± 3
(mmHg)	CH	63 ± 12	60 ± 5	45 ± 6	53 ± 4	51 ± 7	52 ± 2*^#^
	GH	70 ± 5	74 ± 4	68 ± 6	70 ± 3	66 ± 5	67 ± 3

*p < 0.1 compared to CON, ^#^ *p* < 0.05 GH compared to CH

Intracranial pressure: Mean intracranial pressure (ICP) recordings did not show any significant changes during the 2 h time period within or between groups. Calculation of cerebral perfusion pressure (CPP) revealed a lower CPP in the CH cohort compared to CON and GH (Table 2, *p* < 0.05).

**Fig. 2:**
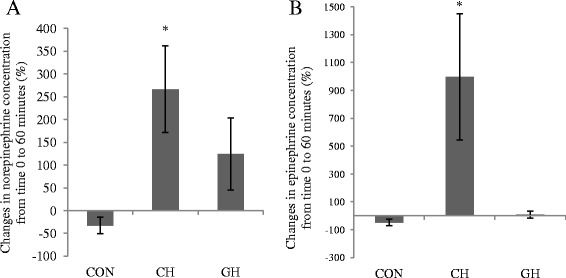
Percent changes in catecholamine concentrations in venous blood extracted from the jugular vein after 60 min of treatment. **a** Percent change of norepinephrine concentrations from baseline to time 60 in CON, CH and GH groups. **b** Percent change of epinephrine concentrations from baseline to time 60 in CON, CH and GH groups. *p < 0.1 compared to CON. Data presented as mean ± SEM

Catecholamines: To assess SNS activity, plasma catecholamine concentrations were measured at baseline and after 60 min of the study. Similar to previous work in which Irwin et al. reported a cerebral hypoxia induced increase in norepinephrine [10], there was a greater norepinephrine (*p* = 0.07) and epinephrine (*p* = 0.10) in CH compared to CON (Fig. 2a-b). Norepinephrine and epinephrine concentrations increased between baseline (time 0) to 60 min in nearly all individual CH and GH canines, while catecholamines remained unchanged in the CON dogs (individual data not shown).

**Fig. 3:**
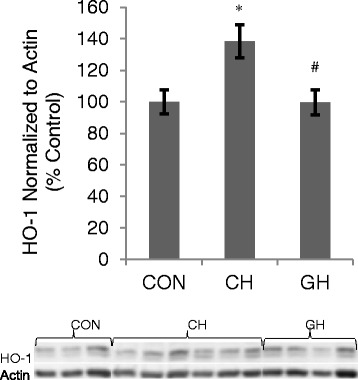
HO-1, phase II antioxidant protein, expression in brain tissue, measured by western blotting. Bar graphs (*top*) reflect the protein content normalized to actin and expressed as % CON while bottom images are representative immunoblots and actin as a loading control protein. *p < 0.05 compared to CON. #*p* < 0.05 compared to CH. Data presented as mean ± SEM

Hypoxia induced oxidative stress: Presence of oxidative stress in lung and brain tissues was evaluated by assessing lipid peroxidation and protein carbonylation. No group differences in these markers of oxidative stress were detectable at the 2-h time point of treatment (data not shown). To assess Nrf2 activation, the nuclear to cytoplasmic ratio of this protein in lung and brain was measured in all three groups. No group differences in the nuclear to cytoplasmic ratio of Nrf2 were detectable at the 2-h time point of treatment (data not shown). To detect whether there was a hypoxia-induced upregulation of ARE-regulated phase II antioxidants, expression of Nrf2, HO-1, NQO1, and SOD1 were measured in lung and brain of all three animal groups. There was no difference in Nrf2 expression in lung or brain, or in lung HO-1 or brain SOD1, across treatment groups (data not shown). Brain HO-1 expression was greater in CH compared to CON and in CH compared to GH (Fig. 3, *p* < 0.05).

## Discussion

Cerebral hypoxia is a common feature of many neuropathologies [23], yet limited data are available on the mechanisms that induce pulmonary edema in the presence of hypoxic brain insults. The current study represents an appreciable advancement of previous methods [9, 10] due to consideration of ICP and controlling for CBF. In the presence of similar CBF, both cerebral and global hypoxia resulted in pulmonary edema. Currently there is no clear evidence of how changes in hemodynamic parameters are involved in the onset of NPE. Hence observing the role of hypoxia, irrespective of changes in blood flow during the generation of edema in lung, was an important additional insight to the previous investigation. The present study suggests that at 2 h of cerebral or global hypoxia there was no detectable oxidative stress, however, our data do suggest involvement of sympathetic activity in induction of pulmonary edema.

Novelty of the model: Several important and novel aspects of the model are noteworthy. This experiment was the first to monitor ICP in the canine model during cerebral hypoxia, allowing for the calculation of CPP. Even though ICP was measured indirectly by probe placement in the subdural space rather than in the ventricles, it allowed us to assess the magnitude of change in ICP. This study was also the first to control CBF during an isolated cerebral hypoxia model by matching CBF during both cerebral and global hypoxia. We found that CBF does not appear to be a major determinant of development of pulmonary edema. That is, pulmonary edema may occur in the absence of changes in CBF, an observation not addressed in our previous study.

Pulmonary hemodynamics: It has been suggested that an increase in PAP contributes to the pathogenesis of NPE [27]. We found that PAP was significantly greater in CH and GH compared to CON. The absence of a greater afterload in pulmonary vasculature in our study was indicated by a lack of differences in Pwedge between the CH and GH compared to CON. Thus we have observed that an increase in PAP can occur in NPE.

Systemic hemodynamics: Sensing of hypoxia occurs through specialized chemoreceptor cells located in bifurcation of carotid artery that regulate cardiovascular and ventilatory rates. Hypoxia, in general, decreases the vascular resistance and CO is subsequently altered so that physiologic MAP is maintained [28]. As shown in the current study, GH led to greater CO compared to CH. Augmented CO in GH group was attributed to an increase in SV since the HR in the GH animals was not different compared to CON and CH. MAP in GH and CON was not significantly different, which implies a CO compensation for hypoxic vasodilation. In our study, the hypoxic perfusate to the brain bypassed the chemoreceptors such that hypoxia was not detected by the chemoreceptors and MAP was significantly attenuated in CH compared to CON. Taken together, systemic compensatory responses to hypoxia were absent in our isolated cerebral hypoxia model.

The dissociation between circulating catecholamines and MAP in CH was unexpected, but is not without precedent. For example, in humans, while breathing hypoxic gas mixtures, sympathetic activation in the absence of vasoconstriction has been documented [29]. Further, in older men with hypertension, 24-h circulating norepinephrine concentration is inversely associated with mean arterial pressure [30]. Finally, during surgery following administration of the anesthetic desfluranes, hemodynamic responses to catecholamines are reversed [31]. Potential mechanisms for reduced and dissociated catecholamine responses include decreased adrenergic receptor sensitivity, increased catecholamine clearance [29], altered catecholamine metabolism, and/or acute septic [32, 33] or hemorrhagic shock [34]. The specific mechanism contributing to increased norepinephrine in the absence of an increase in mean arterial pressure in our study is unclear but warrants further investigation.

SNS activity: Acute hypoxia has been well-established as a sympathetic activator [28]. However, the role of SNS activity in development of NPE in isolated cerebral hypoxia compared to global hypoxia is not known. To evaluate the systemic SNS activity, plasma catecholamine concentrations were quantified. Since catecholamine concentrations are the net result of secretion, spill-over, reuptake, and excretion, comparison of concentrations at baseline and 60 min was performed to better characterize the net change in each canine. Increases in catecholamines, CO, and SV in GH suggest that the physiological chemoreceptor response may be activated through the SNS in this group.

A 6-fold increase in norepinephrine in CH compared to CON suggests that SNS activity may be higher with cerebral hypoxia compared to normoxia. An increase in SNS activity in CH occurred in the presence of increased PAP. Since the hypoxic blood bypassed the carotid chemoreceptors in the CH group such that chemoreceptor “sensing” of hypoxia could not occur, SNS activation in the CH group likely did not ensue through this pathway. Neurons that normally contribute to oxygen sensing are located in the rostral ventrolateral medulla, the caudal hypothalamus, the pre-Bötzinger complex, and the nucleus tractus solitarius. In the absence of a carotid chemoreceptor response, our findings suggest oxygen sensing in these higher centers are involved in SNS activation and the subsequent increase in PAP that contribute to the formation of pulmonary edema. Whether or not other factors are involved in SNS activation will require further investigation.

Role of oxidative stress in NPE: Whether or not oxidative stress contributes to sympathetic activation, increased PAP, or pulmonary edema is unclear. In our study, markers of oxidative stress and antioxidant response were not higher in lung tissue at 2 h of hypoxia. However brain HO-1, a phase II antioxidant downstream of Nrf2, was significantly higher in CH compared to CON. In vitro data from our lab have shown that Nrf2 translocates to the nucleus, an indicator of Nrf2 activation, within 15 min of an oxidative challenge [22]. However, Nrf2 nuclear localization is not sustained as there is a subsequent nuclear export of Nrf2 [35]. Therefore, we were not surprised by the absence of Nrf2 expression in the nucleus after 2 h of hypoxia, yet higher levels of brain HO-1, a Nrf2 downstream protein, in CH compared to CON suggest Nrf2 was activated earlier in the experiment.

Higher levels of brain HO-1 in isolated cerebral hypoxia in the current study suggest that redox sensitive signaling in the brain could contribute to activation of the SNS and induction of pulmonary edema, a finding supported by previous reports [24, 36, 37]. For example, hypoxia acts directly on brainstem neurons, as opposed to only carotid body inputs that are carried within the carotid sinus nerve to the nucleus tractus solitarius and the brainstem respiratory centers [37]. Knowing that hypoxia can induce ROS production [14, 15], it is possible that brain hypoxia activates higher centers of SNS through redox sensitive signaling pathways, which leads to increased PAP and induction of pulmonary edema. Also, in the presence of elevated hypothalamic NADPH oxidase activity (a marker of oxidative stress), the expression level of tyrosine hydroxylase, an enzyme responsible for the synthesis of catecholamines, was higher in the nucleus tractus solitarius of the brain stem [38]. These data, along with previous findings that HO-1 is induced in hypoxia-sensitive brain regions [24, 39], are consistent with our data suggesting that increased expression of HO-1, a compensatory response to oxidative stress, could act as a sensor in brain hypoxia and may have a role in induction of SNS activity.

Limitations of the study: We recognize that all of our hemodynamic measurements were performed in anesthetized animals. We have noted that two out of six animals in CH group had high values of ICP at the baseline. Further PAP baseline values were higher than expected in two animals in CH and GH groups. This could be attributed to the variable response to anesthetics [40]. However, none of the procedures described could have been performed in the absence of anesthesia and thus, we feel this is an acceptable limitation. Several of the differences we report did not attain statistical significance although they may be physiologically significant [41]. Finally, on a technical note, ICP was not determined directly, rather it was estimated from subdural probe placement. This approach is not without precedent, indeed, it has been validated against gold-standard techniques [42].

## Conclusions

In conclusion, the present study suggests that increased SNS activity and PAP, in the absence of CBF alterations, contributes to pulmonary edema in isolated cerebral hypoxia. Furthermore, isolated cerebral hypoxia for 2 h induces HO-1 in the brain, suggesting redox sensitive signaling and a compensatory antioxidant response to hypoxia. In this way, it seems that HO-1 may be an oxygen sensor in the brain, leading to compensatory activation of the SNS, and induction of pulmonary edema through a rise in PAP.

## Abbreviations

NPE: Neurogenic pulmonary edema
ICP: Intracranial pressure
PAP: Pulmonary artery pressure
SNS: Sympathetic nervous system
CPP: Cerebral perfusion pressure
CBF: Cerebral blood flow
HR: Heart rate
CO: Cardiac output
MAP: Mean arterial pressure
ROS: Reactive oxygen species
SV: Stroke volume
Nrf2: Nuclear factor erythroid 2 related factor 2
HO-1: Hemeoxygenase-1
NQO1: NAD (P) H, quinone oxidoreductase 1
SOD1: Superoxide dismutase 1
Pwedge: Pulmonary capillary wedge pressure
LWW/LDW: Lung wet weight to lung dry weight ratio
